# Imaging mass cytometry dataset of small-cell lung cancer tumors and tumor microenvironments

**DOI:** 10.1186/s13104-025-07460-4

**Published:** 2025-09-08

**Authors:** France Rose, Olta Ibruli, Luca Lichius, Martha Kiljan, Gokcen Gozum, Manoela Iannicelli Caiaffa, Jiali Cai, Li-na Niu, Jan M. Herter, Holger Grüll, Reinhard Büttner, Filippo Beleggia, Graziella Bosco, Julie George, Grit S. Herter-Sprie, Hans Christian Reinhardt, Katarzyna Bozek

**Affiliations:** 1 Center for Molecular Medicine Cologne, Faculty of Medicine, University Hospital Cologne, University of Cologne, Cologne, Germany; 2 Institute for Biomedical Informatics, Faculty of Medicine, University Hospital Cologne, University of Cologne, Cologne, Germany; 3 Department of Translational Genomics, Faculty of Medicine, University Hospital Cologne, University of Cologne, Cologne, Germany; 4 Department I of Internal Medicine, Faculty of Medicine, University Hospital Cologne, University of Cologne, Cologne, Germany; 5 Mildred Scheel School of Oncology Aachen Bonn Cologne Düsseldorf (MSSO ABCD), Faculty of Medicine, University Hospital Cologne, University of Cologne, Cologne, Germany; 6 Department II of Internal Medicine, Faculty of Medicine, University Hospital Cologne, University of Cologne, Cologne, Germany; 7 Department of Radiation Oncology and Cyberknife Center, Faculty of Medicine, University Hospital of Cologne, University of Cologne, Cologne, Germany; 8 Department of Radiation Oncology, MVZ Prof. Dr. Uhlenbrock und Partner, Gesundheitscampus Josephs-Hospital, Warendorf, Germany; 9 Institute for Diagnostic and Interventional Radiology, Faculty of Medicine, University Hospital Cologne, University of Cologne, Cologne, Germany; 10 Institute of Pathology, Faculty of Medicine, University Hospital Cologne, University of Cologne, Cologne, Germany; 11 Department of Otorhinolaryngology, Faculty of Medicine, University Hospital of Cologne, University of Cologne, Cologne, Germany; 12 Department of Hematology and Stem Cell Transplantation, Germany 19 Center for Molecular Biotechnology, University Hospital Essen, Essen, Germany; 13 West German Cancer Center, University Hospital Essen, Essen, Germany; 14 Germany 20 Department of Medical Oncology, Fachklinik Hornheide, Münster, Germany; 15 DKTK Partner Site Essen/Düsseldorf, University Hospital Essen, Essen, Germany; 16 Center for Molecular Biotechnology, University Hospital Essen, Essen, Germany

**Keywords:** SCLC, IMC, MIBI-TOF, Hyperion, Tumor microenvironment, Mouse models

## Abstract

**Objectives:**

Small cell lung cancer (SCLC) accounts for approximately 15% of lung tumors and is marked by aggressive growth and early metastatic spread. In this study, we used two SCLC mouse models with differing tumor mutation burdens (TMB). To investigate tumor composition, spatial architecture, and interactions with the surrounding microenvironment, we acquired multiplexed images of mouse lung tumors using imaging mass cytometry (IMC). These data build upon a previously published characterization of the mouse model.

**Data description:**

After tumor detection, mice were assigned to one of five treatment groups. Lung tumor tissues were imaged with a 37-marker IMC panel designed to identify major cell types—tumor, immune, and structural—as well as their functional states. When possible, each tumor was sampled both at its center and border regions. Tumor masks in the form of binary images are provided to delineate tumor areas. Additional metadata include tumor onset and endpoint dates to support downstream correlation or predictive analyses based on the image data. This dataset offers a valuable resource for studying the histological and cellular complexity of SCLC in a genetically controlled mouse model across multiple therapeutic conditions.

## Objective

Small cell lung cancer (SCLC) remains one of the most lethal forms of lung cancer. Although initial responses to chemotherapy are often strong, most patients ultimately relapse, and overall survival gains brought by immune checkpoint inhibitors (ICI) have been limited [1, 2]. Progress in developing more effective treatments has been hampered in part by preclinical models not fully capturing the disease’s complexity. The widely used RP mouse model, characterized by the loss of *Rb1* and *Trp53*, fails to capture the full molecular heterogeneity and mismatch repair (MMR) deficiencies now identified in subsets of SCLC patients [3, 4]. Recently a novel SCLC mouse model capturing MMR deficiency, called the RPM model, was introduced [5]. Here, we report the first tissue-level imaging of SCLC tumors in RPM model, alongside with the classical RP model.

The characterization of this new mouse line was conducted at the macro scale under five different lines of treatments (Platinum-based chemotherapy; Immune checkpoint inhibition; Treg suppressor; Combination of chemotherapy and immune checkpoint inhibition; Combination of Treg suppressor with chemotherapy and immune checkpoint inhibition; Vehicle control) [5]. The following key differences were observed: 1/ RPM tumors exhibited MMR deficiency, a high tumor mutational burden (TMB), and an elevated load of candidate neoantigens compared to RP lesions, suggesting enhanced immunogenicity; 2/ The time from tumor manifestation to death was substantially shortened for animals with MMR-defective tumors, indicating increased aggressiveness; and 3/ The overall survival of RPM animals was significantly improved when exposed to ICI.

As a follow-up study, tissues from this cohort were collected and imaged with a high-multiplexed panel to uncover tumor heterogeneity and spatial characteristics. The objective was to further characterize this new RPM model and its responses to treatments. Unfortunately, the project ran out of funding before an in-depth analysis of the multiplexed image data could be carried out.

## Data description

The dataset comprises 221 regions of interest (ROIs) imaged from two frozen tissue microarrays (TMAs) sections stained with metal-coupled antibodies and imaged via Imaging Mass Cytometry (IMC) [6]. One TMA contains all tissues from the RP mice, the other from the RPM mice. Each TMA block contains cores (1.5 mm in diameter) from mouse lung tumor samples and additional tissues for control (spleen, lymph node). For each mouse tumor sample, two areas were selected for imaging with the help of the IMC software viewer: one located in the central tumor region and one at the tumor-normal lung border (see metadata file *1*).

A 37-marker panel was employed (see metadata file *2*). The panel includes cellular markers for tumor cells, stromal compartments (endothelial cells, fibroblasts), and various immune cell types (T cells, B cells, macrophages, etc.). The panel also includes functional markers that assess cellular states like activation, exhaustion, and proliferation. This panel was optimized to minimize overlap and enhance the specificity of antibody binding based on prior samples. Control murine spleen samples were utilized to validate antibody specificity and sensitivity. The high number of simultaneous markers enables a detailed description of cellular content as well as spatial relationships between cells.

Each ROI is paired with a binary tumor mask (manually delineated tumor regions using normalized RGB composites DNA [blue], NCAM [red], and PD-L1 [green] via the Fiji software), allowing for comparisons of immune and cellular profiles between tumor interiors and tumor-normal boundaries. Metadata link each ROI to the mouse line, tissue type, treatment groups, dates of tumor onset, and mouse termination dates (see metadata file *1*). From the original .mcd file format obtained from the IMC device, zarr ome files were extracted, discarding background channels only (see files in the RP-TMA and RPM-TMA Data sets, cf. Table 1; each zarr ome contains all 37 channels for each ROI and the tumor mask when the tissue was extracted from the lung). Furthermore, some ROIs were excluded if the imaged area was too small (5 excluded ROIs) or the staining quality was too low (7 excluded ROIs; the list of discarded ROIs is in the Study description file).

No further processing (e.g. signal processing, segmentation) was conducted, as the empty metadata file 3 shows, leaving the rest of the analysis pipeline up to the users Table 1.

**Table 1:** Overview of data files/data sets

Label	Name of data file/data set	File types (file extension)	Data repository and identifier (DOI or accession number)
*Study description*	*idr0170-study.txt*	*Text (.txt)*	Image Data Resource DOI: https://doi.org/10.17867/10000208 [6]
*Metadata File 1*	*idr0170-experimentA-assays.tsv*	*Table (.tsv)*	Image Data Resource DOI: https://doi.org/10.17867/10000208 [6]
*Metadata File 2*	*idr0170-experimentA-IMCchannels.tsv*	*Table (.tsv)*	Image Data Resource DOI: https://doi.org/10.17867/10000208 [6]
*Metadata File 3*	*idr0170-experimentA-processed.tsv*	*Table (.tsv)*	Image Data Resource DOI: https://doi.org/10.17867/10000208 [6]
*Data set 1*	*RP-TMA*	*Ome zarr files (.ome.zarr)*	Image Data Resource DOI: https://doi.org/10.17867/10000208 [6]
*Data set 2*	*RPM-TMA*	*Ome zarr files (.ome.zarr)*	Image Data Resource DOI: https://doi.org/10.17867/10000208 [6]

## Limitations

The spatial resolution of IMC is fixed at 1 μm² in the xy-plane, as the technology relies on a laser to ablate successive small tissue regions, with the spatial layout reconstructed from these discrete measurements.

SCLC tumors are known for their high heterogeneity: variations in tumor architecture, immune cell infiltration, and rare cell populations (e.g., specific immune subsets) can be significant across different regions [7, 8]. The high cost and time constraints associated with IMC imaging necessitated selective sampling of TMA cores, resulting in incomplete spatial coverage of the tissue arrays. Given the limited sample size (numbers of mice per treatment vary from 9 – vehicle RPM mice – to 23; this information for each group of mice is retrievable from metadata file 1), such heterogeneity could hinder robust conclusions and reduce the statistical power of analyses.

Additionally, not all markers exhibited optimal staining quality, as noted in the metadata file 2, which may introduce variability or reduce the reliability of specific protein measurements.

These limitations underscore the need for careful experimental design, validation against established studies on SCLC heterogeneity, and cautious interpretation of results, particularly concerning rare or low-signal markers.

## Data Availability

The data described in this Data note will be freely and openly accessed on Image Data Resource (https://idr.openmicroscopy.org) under accession number idr0170, under the DOI: https://doi.org/10.17867/10000208 [6]. Please see Table 1 for details.

## Abbreviations

ICI: Immune Checkpoint Inhibitor
IMC: Imaging Mass Cytometry
MIBI-TOF: Multiplexed Ion Beam Imaging by Time Of Flight
MMR: Mismatch Repair
ROI: Region of Interest
RP: Rb1^fl/fl^;Trp53^fl/fl^
RPM: Rb1^fl/fl^;Trp53^fl/fl^;Msh2^fl/fl^
SCLC: Small Cell Lung Cancer
TMA: Tissue Microarray
